# Codon harmonization reduces amino acid misincorporation in bacterially expressed *P. falciparum* proteins and improves their immunogenicity

**DOI:** 10.1186/s13568-019-0890-6

**Published:** 2019-10-19

**Authors:** Neeraja Punde, Jennifer Kooken, Dagmar Leary, Patricia M. Legler, Evelina Angov

**Affiliations:** 1 Malaria Biologics Branch, Walter Reed Army Institute of Research, Silver Spring, MD 20910 USA; 2 Naval Research Laboratories, 4555 Overlook Ave, Washington, DC 20375 USA; 3 The Geneva Foundation, 917 Pacific Avenue, Tacoma, WA 98402 USA

**Keywords:** *Pf*CelTOS, Codon-harmonization, Protein translation, Amino-acid misincorporation, Secondary structure, Alpha-helical content

## Abstract

Codon usage frequency influences protein structure and function. The frequency with which codons are used potentially impacts primary, secondary and tertiary protein structure. Poor expression, loss of function, insolubility, or truncation can result from species-specific differences in codon usage. “Codon harmonization” more closely aligns native codon usage frequencies with those of the expression host particularly within putative inter-domain segments where slower rates of translation may play a role in protein folding. Heterologous expression of *Plasmodium falciparum* genes in *Escherichia coli* has been a challenge due to their AT-rich codon bias and the highly repetitive DNA sequences. Here, codon harmonization was applied to the malarial antigen, CelTOS (*Cel*l-*t*raversal protein for *o*okinetes and *s*porozoites). CelTOS is a highly conserved *P. falciparum* protein involved in cellular traversal through mosquito and vertebrate host cells. It reversibly refolds after thermal denaturation making it a desirable malarial vaccine candidate. Protein expressed in *E. coli* from a codon harmonized sequence of *P. falciparum* CelTOS (CH-*Pf*CelTOS) was compared with protein expressed from the native codon sequence (N-*Pf*CelTOS) to assess the impact of codon usage on protein expression levels, solubility, yield, stability, structural integrity, recognition with CelTOS-specific mAbs and immunogenicity in mice. While the translated proteins were expected to be identical, the translated products produced from the codon-harmonized sequence differed in helical content and showed a smaller distribution of polypeptides in mass spectra indicating lower heterogeneity of the codon harmonized version and fewer amino acid misincorporations. Substitutions of hydrophobic-to-hydrophobic amino acid were observed more commonly than any other. CH-*Pf*CelTOS induced significantly higher antibody levels compared with N-*Pf*CelTOS; however, no significant differences in either IFN-γ or IL-4 cellular responses were detected between the two antigens.

## Introduction

*Escherichia coli* expression systems have been widely used for the expression and manufacturing of various malarial antigens owing to their ease of use and advantages in cost and scale despite protein expression and folding obstacles. Common causes cited for poor expression of recombinant genes in heterologous hosts are the species-specific disparities in codon usage. Codon usage frequencies can potentially impact a protein’s function, solubility, and length (Khan et al. 2012).

In *E. coli*, protein folding can occur “co-translationally” at the ribosome (Komar 2009; Kramer et al. 2009; Nissley et al. 2016) and variable translation rates are thought to affect tertiary structure (Nissley et al. 2016). A recent study using genome-wide analysis provided evidence of evolutionary selection for co-translational folding, underscoring the importance of translation kinetics in protein folding (Jacobs and Shakhnovich 2017). More frequently used codons are often found in well-ordered structural elements such as alpha helices, while low usage frequency codons often occur within link/end segments (Thanaraj and Argos 1996). These observations suggest that codon usage frequency plays an inherent role in co-translational folding.

Based on these concepts, we developed a strategy to “recode” target gene sequences for heterologous expression by substituting native codons with synonymous alternates with identical or similar usage frequencies in the expression host. This approach has been termed “codon harmonization”, and applies a two-pronged approach. First, “best fit” codon usage frequency of the native gene is applied to that of the heterologous host. Second, putative link/end segments are identified and recoded to re-establish regions benefitted by slower translation (Angov et al. 2008).

We previously reported single base changes (i.e., FMP003 protein) for synonymous codon replacement can increase soluble protein yields by a factor of approximately ten, compared with native sequence yields (Angov et al. 2008). This protein was produced under cGMP conditions resulting in a highly immunogenic and efficacious product tested against malaria challenge in an Aotus monkey study (Darko et al. 2005). “Harmonizing” all of the codons throughout the gene sequence (i.e., FMP010 protein) yielded an additional 60-fold increase in expression level (Angov et al. 2008). Furthermore, application of this approach to alternative alleles of MSP1_42_ protein, yielded similar high levels of expression. The successes achieved are notable, because some native *P. falciparum* gene sequences expressed in *E. coli* yield no or low yields of recombinant protein (Angov et al. 2008). Here we applied codon harmonization to improve protein expression, yield, and quality of a novel malaria vaccine candidate, *Pf*CelTOS.

“Cell-traversal protein for ookinetes and sporozoites” (CelTOS) is an essential protein in malaria parasites that is required for cell traversal in both mammalian and insect hosts (Kariu et al. 2006). In mice, recombinant *Pf*CelTOS in Montanide ISA 720 elicited potent humoral and cellular immune responses as well as sterile protection against heterologous challenge with *Plasmodium berghei* sporozoites (Bergmann-Leitner et al. 2010). This was corroborated using an alternative recombinant *Pf*CelTOS in glucopyranosyl lipid adjuvant-stable emulsion (GLA-SE) or glucopyranosyl lipid adjuvant-liposome-QS21 (GLA-LSQ) adjuvant (Espinosa et al. 2017). In addition, monoclonal antibodies raised against *Pf*CelTOS inhibited oocyst development of *P. falciparum*, and *P. berghei* expressing *Pf*CelTOS, in *Anopheles gambiae* mosquitoes. Notwithstanding these findings, CelTOS is an attractive target for immunization as it is conserved across plasmodial species (Kariu et al. 2006).

We developed a recombinant protein vaccine candidate based on *Pf*CelTOS in *E. coli*. On characterization of the protein product, we observed that the *Pf*CelTOS reversibly refolds after thermal denaturation; this property may be valuable for cold-chain storage or use in temperate climates. To address the impact of codon harmonization on primary, secondary, and tertiary structure, recombinant protein was produced using the native gene sequence (N-*Pf*CelTOS) and compared with protein produced from the codon-harmonized sequence (CH-*Pf*CelTOS). We utilized circular dichroism (CD), mass spectrometry (MS), and size-exclusion chromatography to characterize the two proteins and identified differences in mass and heterogeneity. A deeper analysis by liquid chromatography–tandem mass spectrometry (LC–MS/MS) revealed amino acid misincorporations in both proteins. Interestingly, despite these changes in amino acid sequence, both proteins reversibly refolded after heat denaturation despite detectable changes in primary and secondary structure. The potential impact of these changes as immunogens also was evaluated in vivo in Balb/cJ mice. Antibody fine specificities were assessed against full length *Pf*CelTOS or subunit fragments reflecting the N-terminus or C-terminus to better define any differences. CH-*Pf*CelTOS induced significantly higher antibody levels compared with N-*Pf*CelTOS; however, no significant differences in cellular responses were detected.

## Materials and methods

### Sequences

Proteins were produced in *E. coli* using either the native or codon harmonized DNA sequences. Gene inserts were synthesized and cloned into the pET(K) expression plasmids (DNA 2.0, currently ATUM, Newark, CA) and transformed into B834 (DE3) *E. coli*. Both native and codon harmonized sequences (GenBank Accession # KH833194) encoded the same 174 amino acids and included a 16 amino acid linker containing an N-terminal 6-Histidine tag (Bergmann-Leitner et al. 2010). A Histidine tag-free *Pf*CelTOS was expressed in *E. coli* as above, and similar to the N- and CH-*Pf*CelTOS proteins were expressed without the native *Pf*CelTOS signal sequence. Primer pairs used to generate the Histidine tag free *Pf*CelTOS clone from the CH-*Pf*CelTOS (N-terminal Histidine tagged protein) used *Xba*I and *Kpn*I to replace the nucleotide sequences using *Xba*I–*Nde*I *Kpn*I 5′-CTAGAAATAATTTTGTTTAACTTTAAGAAGGAGATATACATATGGGTAC-3′ and *Kpn*I *Nde*I–*Xba*I 5′-CCATATGTATATCTCCTTCTTAAAGTTAAACAAAATTATTT-3′ annealed primers. His-tag free PfCelTOS was 161 amino acids long, including two non-native amino acid residues introduced by cloning, GT. All full length clones were initiated at amino acids F R G… and contained 158 of *Pf*CelTOS amino acids. An N-terminal protein fragment of *Pf*CelTOS (natural residues numbering #25-149) and C-terminal fragment of *Pf*CelTOS (residues #85-182) were expressed under identical conditions as for the full length *Pf*CelTOS and used as a reagents to assess fine specificities of immune responses.

### Expression of N-*Pf*CelTOS and CH-*Pf*CelTOS

To investigate the effect of codon usage on *Pf*CelTOS expression, cultures were grown in the presence of 40 µg/mL kanamycin (Sigma Aldrich, St. Louis, MO) in 1 L Difco Terrific Broth (BD Biosciences, San Jose, CA) at 30 °C. Cells were induced by adding 0.1 mM isopropyl β-d-1-thiogalactopyranoside (IPTG) (Sigma Aldrich) at an OD_600_ ~ 0.8–1.0 for protein induction. Cell samples were collected every hour from the time of induction for 3 consecutive hours for analysis by SDS-PAGE (Invitrogen, Waltham, MA). Subunit fragments representing the N-terminus and C-terminus of *Pf*CelTOS essentially were expressed using the same conditions as for full length *Pf*CelTOS.

### Solubility of N-*Pf*CelTOS and CH-*Pf*CelTOS

Cell paste (3 g) was homogenized (Ultra Turrax T-25, Cole Palmer, Vernon Hills, IL) in 60 mL of lysis buffer (PBS; pH 7.4) (Quality Biological, Gaithersburg, MD). Cells were subjected to microfluidization (Microfluidics Corporation, Model M-110 Y, Westwood, MA) and the cell lysates were divided equally into four parts (by volume). Each part was treated with 1% Tween 80 (v/v) (Fisher Scientific), 1% deoxycholate (v/v) (Fisher Scientific, Rockville, MD) or 1% sarkosyl (v/v) (Sigma-Aldrich, St. Louis, MO) and one part was left “untreated”. Detergent extractions were carried out at 30 °C for 1 h in an incubator shaker at ~ 50 rpm. Treated lysates were centrifuged at 12,000 rpm for 1 h at 4 °C to separate soluble supernatants and insoluble pellet fractions. Samples from each fraction were prepared for SDS-PAGE/Coomassie Blue staining (Bio-Rad, Philadelphia, PA).

### Purification of N-*Pf*CelTOS and CH-*Pf*CelTOS

Cell paste (4 g) for both clones (N-*Pf*CelTOS and CH-*Pf*CelTOS) was homogenized in 60 mL of lysis buffer (10 mM NaH_2_PO_4_, 50 mM NaCl, 10 mM imidazole, 2 mM MgCl_2_, pH 7.4) and lysed by microfluidization. To adjust the final salt concentration, 5 M NaCl was added to each lysate. Protein was extracted at 30 °C for 30 min by addition of 1% (v/v) sarkosyl. Extracted lysates were centrifuged at 12,000 rpm at 4 °C for 1 h to isolate soluble proteins.

Purification for both proteins was carried out simultaneously under identical conditions. The lysates were passed through 3.5 mL Nickel-nitriletriacetic acid (Qiagen, Germantown, MD) gravity columns. Columns were washed with 20 column volumes (CV) of equilibrium buffer (10 mM NaH_2_PO_4_, 1 M NaCl, 0.3% sarkosyl, 10 mM imidazole, pH 7.4), 15CV of wash buffer 1 (10 mM NaH_2_PO_4_, 500 mM NaCl, 0.3% sarkosyl, pH 6.5), 10CV of wash buffer 2 (10 mM NaH_2_PO_4_, 200 mM NaCl, pH 6.5), and 10CV of wash buffer 3 (10 mM NaH_2_PO_4_, 50 mM NaCl, 100 mM imidazole, pH 6.2). Proteins were eluted with elution buffer, (10 mM NaH_2_PO_4_, 50 mM NaCl, 300 mM imidazole, pH 6.2) and dialyzed against phosphate buffer saline (PBS) (pH 7.4) at 4 °C. Dialyzed proteins were polished through 3.5 mL of Q-Sepharose (GE Healthcare, Chicago, IL) and the final product dialyzed into PBS (pH 7.4). The N-terminal protein fragment of *Pf*CelTOS (residue #25-149) and C-terminal fragment of *Pf*CelTOS (residue #85-182) essentially were purified using identical chromatographic conditions as the full length *Pf*CelTOS.

For the purification of histidine tag-free CH-*Pf*CelTOS, lysate was passed through a 2 mL Q-Sepharose column followed by 30CV of equilibration wash (10 mM NaH_2_PO_4_, 50 mM NaCl, 0.2% Tween 80; pH 7). The column was washed with 40CV of wash 1 buffer (10 mM NaH_2_PO_4_, 260 mM NaCl, 0.2% Tween 80; pH 7) followed by elution with 10CV of elution buffer (10 mM NaH_2_PO_4_, 500 mM NaCl, 0.2% Tween 80; pH 7). Eluted fraction was dialyzed in Q-Sepharose equilibration buffer (10 mM NaH_2_PO_4_, 50 mM NaCl, 0.6% beta octyl-glucopyranoside) and was loaded onto a 2 mL Q-Sepharose column. Protein was eluted using a linear salt gradient formed between the equilibration buffer and elution buffer (10 mM NaH_2_PO_4_, 500 mM NaCl, 0.6% beta β-octyl-glucopyranoside). The eluted fraction was dialyzed into HIC equilibration buffer, (50 mM NaH_2_PO_4_, 1 M ammonium sulfate, pH 7.4), and loaded onto a 1 mL Phenyl Sepharose (GE Healthcare, Chicago, IL) and eluted using a linear gradient formed between the equilibration buffer and elution buffer (50 mM NaH_2_PO4, 50 mM ammonium sulfate, pH 7.4). The final elution fraction was dialyzed in 1× PBS, pH 7.4.

### SDS-PAGE/Coomassie Blue and immunoblotting

Purified proteins were separated on 4–20% gradient Tris–glycine SDS-PAGE gels and stained with Coomassie Blue R-250. For western blotting, proteins were transferred to 0.2 µm nitrocellulose membranes (Invitrogen, Waltham, MA) and blocked in PBS (pH 7.4), 0.1% Tween 20 (PBS-T) with 0.5% non-fat dry milk for 30 min at room temperature (RT). Western blots were probed with *Pf*CelTOS-specific mouse mAbs 3D11.D4, 4H9.C3 and 3C3.B3 and anti-Histidine mouse mAb (at 1:3000 each) (Takara Bio, Mountain View, CA) for 1 h. After washing with PBS-T, blots were probed with alkaline phosphatase (AP)-conjugated anti-mouse IgG (1:5000) (Southern Biotech, Birmingham, AL). Blots were washed with PBS-T and developed for 10 min at RT with 4-nitro-blue tetrazolium chloride (NBT) and 5-bromo-4-chloro-3-indolyl phosphate (BCIP) (Roche, Branchburg, NJ) in 0.1 M sodium chloride, 0.005 M magnesium chloride and 0.1 M Tris–HCl pH 9.

### Stability at different temperatures

Purified N-*Pf*CelTOS and CH-*Pf*CelTOS were subjected to stability analysis at different temperatures. A 1 μg/10 μL aliquot of each protein was incubated at either 37 °C or 65 °C for 1, 4 and 24 h. Samples were analyzed by SDS-PAGE/Coomassie Blue staining and western blotting.

### Membrane lipid strip assay

Membrane lipid strips (Echelon Biosciences Inc., Salt Lake City, UT) were blocked in blocking buffer (pH 7.2) 1× PBS, 0.1% Tween 20, 3% bovine serum albumin (BSA) for 1 h. The strips were probed with 2 µg/mL of N- and CH-*Pf*CelTOS for 1 h. The proteins were pre-treated at either 37 °C or 65 °C for 1, 4 and 24 h. The strips were washed three times with 5 mL of wash buffer (1× PBS; 0.1% Tween 20) at 5 min intervals and then probed with *Pf*CelTOS-specific rabbit polyclonal serum diluted in blocking buffer (1:5000) for 1 h as the primary antibody and anti-mouse HRP conjugate (1:5000) (KPL, Gaithersburg, MD) as the secondary antibody. The strips were developed with Pierce ECL Western Blotting detection kit (Thermo Scientific, Rockford, IL) for 1 min and imaged using VersaDoc (Bio-rad, Hercules, CA). Temperature-untreated proteins (T0) were used as the binding controls for the membrane lipid strip assay.

### Circular dichroism spectroscopy

Thermal denaturation was monitored (2 °C per minute) from 20 to 95 °C using a Jasco 810 circular dichroism spectropolarimeter (Jasco Inc., Japan) fitted with a Peltier temperature control unit. The melting temperature was determined from a four-parameter fit of the ellipticity at 220 nm. A protein concentration of 13 μM (N-*Pf*CelTOS) and 10 μM (CH-*Pf*CelTOS) and a 1 mm cuvette was used for CD analysis. Machine units were converted to molar ellipticity to account for differences in protein concentrations.

### Mass spectrometry and peptide analysis

Proteins in 200 mM ammonium bicarbonate were directly injected into a triple-TOF 5600 high resolution mass spectrometer (Sciex, Foster City, CA), full protein TOF spectra was acquired using Analyst TF software (Version 1.7, Sciex, Foster City, CA). Spectra were analyzed and overlaid using Peakview software (Sciex, Foster City, CA). Molecular weights were calculated from spectra using a BioToolKit application for Peakview software. Calculated MW = 19.027.03 g/mol, pI = 5.15, and the ɛ = 9970 M^−1^ cm^−1^ for each protein, N-*Pf*CelTOS and CH-*Pf*CelTOS.

### LC–MS/MS

#### Trypsin digest/peptide extraction

Proteins were run on 4–20% Tris–glycine Invitrogen gels. Protein bands were cut from the gel and placed in individual 1.5 µL tubes with 300 µL 50% acetonitrile (ACN) in 25 mM ammonium bicarbonate until fully destained followed by alkylation in 30 µL 50 mM iodoacetamide for 30 min at RT. Bands were washed with 500 µL of 100 mM ammonium bicarbonate for 10 min and dehydrated in 600 µL 100% ACN followed by drying for 3 min in a SpeedVac. Gel bands were treated with 8 µL of 1 µg/µL trypsin and 292 µL of 50 mM ammonium bicarbonate. Samples were incubated at 37 °C for 15–18 h, at 450 rpm. Reactions were stopped by addition of 2 µL of 5% formic acid followed by addition of 100 µL HPLC-grade water. Samples were allowed to incubate at RT for 10 min followed by centrifugation for 5 min at 13,000 rpm. Supernatant was aliquoted in labeled tubes containing extraction solution (5% of 50% ACN, 5% formic acid). Peptides were extracted by adding 400 µL of extraction solution, vortexing and allowing to sit for 15 min followed by centrifuging for 5 min; extraction was repeated three times.

#### Solid phase extraction

Samples were desalted using OASIS HLB 1 cc (30 mg) reversed phase cartridges (waters). Columns were activated with 1000 µL of 0.1% trifluoroacetic acid (TFA), 80% ACN twice. Columns were washed with 1000 µL of 0.1% TFA, 5% ACN twice. Samples were collected in 1.5 mL lo-bind tubes. 1000 µL of sample was added to each column. Peptides were eluted with 1000 µL of 0.1% TFA, 80% ACN.

#### LC–MS

Samples were reconstituted with 10 µL; 0.1% formic acid and analyzed on Ultimate 3000 RSLCnano (Thermo Fisher) system in conjunction with Orbitrap Lumos Fusion Mass Spectrometer (Thermo Fisher).

### Size-exclusion chromatography

PBS and the gel-filtration column calibration standards were purchased from Sigma. PD-10 and Superdex G-200 columns were purchased from G.E. Healthcare. A Superdex G-200 column was equilibrated with PBS, pH 7.4. The column was first calibrated using three protein standards and a 0.5 mL loading loop (0.5 mL/min, 4 °C). A calibration curve was made by plotting the calculated MW vs. the elution volume corresponding to the peak max. The data was fit to a single exponential decay equation using Grafit 5.0.13 (Erithacus Software Limited). Each protein sample was run in the same fashion. Three proteins were loaded: (1) N-*Pf*CelTOS; (2) CH-*Pf*CelTOS; (3) tag-free CH-*Pf*CelTOS.

### N-*Pf*CelTOS and CH-*Pf*CelTOS mouse immunogenicity

Six to seven week-old female Balb/cJ mice (The Jackson Laboratories, Sacramento, CA) were purchased and housed under pathogen-free conditions. Ten mice per group were immunized three times on a 3 week interval by the intramuscular route in the thigh muscle with 10 µg N-*Pf*CelTOS/Montanide ISA-720 (Seppic Inc. New Jersey, NY) or 10 µg CH-*Pf*CelTOS/Montanide ISA-720 in 100 µL; 50 µL per side. Blood samples were collected before every immunization for evaluating humoral responses. Two weeks after the third immunization splenocytes were collected for evaluating cellular responses. An adjuvant control group, mice vaccinated with ISA 720 were shared with a concurrent study.

### ELISA

Blood samples were collected from lateral tail veins prior to every immunization. *Pf*CelTOS-specific antibodies were analyzed by enzyme-linked immunosorbent assay (ELISA). Briefly, 2HB Immulon plates (Thermo Scientific, Rochester, NY) were coated with 100 µL/well of codon-harmonized of each 25 ng *Pf*CelTOS, 15 ng N-terminal *Pf*CelTOS or 15 ng C-terminal *Pf*CelTOS in PBS, pH7.4 (Quality Biological, Gaithersburg, MD) and incubated overnight at 4 °C in a humidified chamber. After blocking with PBS, 1% BSA at 22 °C (VWR, Chicago, IL) for 1 h, individual samples prepared at single dilutions were added to the plate. Antibody concentration was determined by establishing a standard curve (run in parallel with each assay) with purified mouse IgG. For each serum tested, we determined a concentration that was within the linear portion of the reaction curve and used this dilution to extrapolate the actual antibody concentration in the assay wells. A mouse-IgG (Invitrogen, Rochester, NY) standard curve was run in tandem. Plates were incubated for 2 h at 37 °C in a humidity chamber followed by addition of 100 µL/well AP-conjugated anti-mouse (Promega, Madison, WI). The plates were incubated at 22 °C in a humidity chamber for 1 h followed by addition of Blue Phos substrate (Sera Care, KPL, Gaithersburg, MD). Development was arrested by addition of 2× AP Stop solution (Sera Care, KPL) after 15 min. The plates were read at an absorbance of 630 nm on SpectraMax M2 (Molecular Devices, Downingtown, PA). The concentration of *Pf*CelTOS-specific (full-length, N or C terminal) antibodies (µg/mL) was calculated from the linear portion of the mouse-IgG standard curve.

### ELISpot

Cellular responses were evaluated using IFN-γ and IL-4 enzyme-linked immunospot assays (ELISpot). Spleens were harvested under sterile conditions 2 weeks post-third immunization and splenocytes were isolated. The splenocytes were suspended in 90% dimethyl sulfoxide (DMSO, Sigma Aldrich, St. Louis, MO); 10% fetal bovine serum (FBS, Gibco, Rockville, MD) and stored under liquid nitrogen until testing. Multiscreen plates were coated with IFN-γ and IL-4 capture antibodies (ELISpot, R&D Systems, Minneapolis, MN) in sterile PBS, pH 7.4 (Quality Biological, Gaithersburg, MD) according to the manufacturer’s instructions and incubated overnight at 4 °C in a humidified chamber. The plates were washed with DMEM (Quality Biological, Gaithersburg, MD) followed by blocking with complete media containing DMEM; 10% FBS; 2 mM l-glutamine, 58,000 units penicillin/58,000 µg streptomycin, 10 mM HEPES; MEM NEAA, 1 mM sodium pyruvate, 2-mercaptoethanol. Plates were coated with splenocytes from individual samples at a concentration of 2 × 10^5^ cells per well. Cells were stimulated with 10 µg/mL *Pf*CelTOS, 6 µg/mL N-term *Pf*CelTOS, 6 µg/mL C-term *Pf*CelTOS and 1 µg/mL *Pf*CelTOS 15-mer peptide pool for 48 h. Secreted cytokines were detected according to the manufacturer’s instructions. Spots were counted using the ELISpot reader (Autoimmun Diagnostika, Straβberg, Germany).

### Statistical analysis

Statistical significance of serological and cellular responses where p < 0.05 is considered significant, were evaluated using parametric two-tailed, unpaired T-tests and multiple T-tests, respectively (GraphPad Prism, v 6.07, San Diego, CA). For multiple T-tests, statistical significance was determined using the Holm-Sidak method.

## Results

### Features of the expressed proteins

Previously, we observed a significant increase in the expression of MSP1_42_-FVO proteins from codon harmonized sequences (Khan et al. 2012). Protein expressed from either a native or a codon harmonized gene sequence of *Pf*CelTOS expressed well in *E. coli* (data not shown). However, the average yield using an identical purification process for N-*Pf*CelTOS and CH-*Pf*CelTOS was 1.35 and 2.57 mg/g of wet cell paste, respectively, approximately twofold higher than for the CH sequence. Protein solubility is an important property of recombinant proteins and is dependent on the pH and pI of a protein, ionic strength, temperature, the presence of various solvent additives, and the amino acids on the protein surface. Based on amino acid sequence, *Pf*CelTOS is predicted to be highly soluble. PRO-Sol analysis predicted a scaled solubility value of 0.864 (pI = 5.21) which is higher than the population average of soluble *E. coli* proteins with a scaled solubility value of 0.45 (Hebditch et al. 2017). Experimentally, both *Pf*CelTOS proteins partitioned into the soluble phase after lysis in phosphate buffered saline (PBS), pH 7.4 under all detergent treatment conditions (1% v/v each, Tween 80, β-octyl-glucopyranoside, sarkosyl), as well as in the absence of detergent (data not shown).

**Fig. 1:**
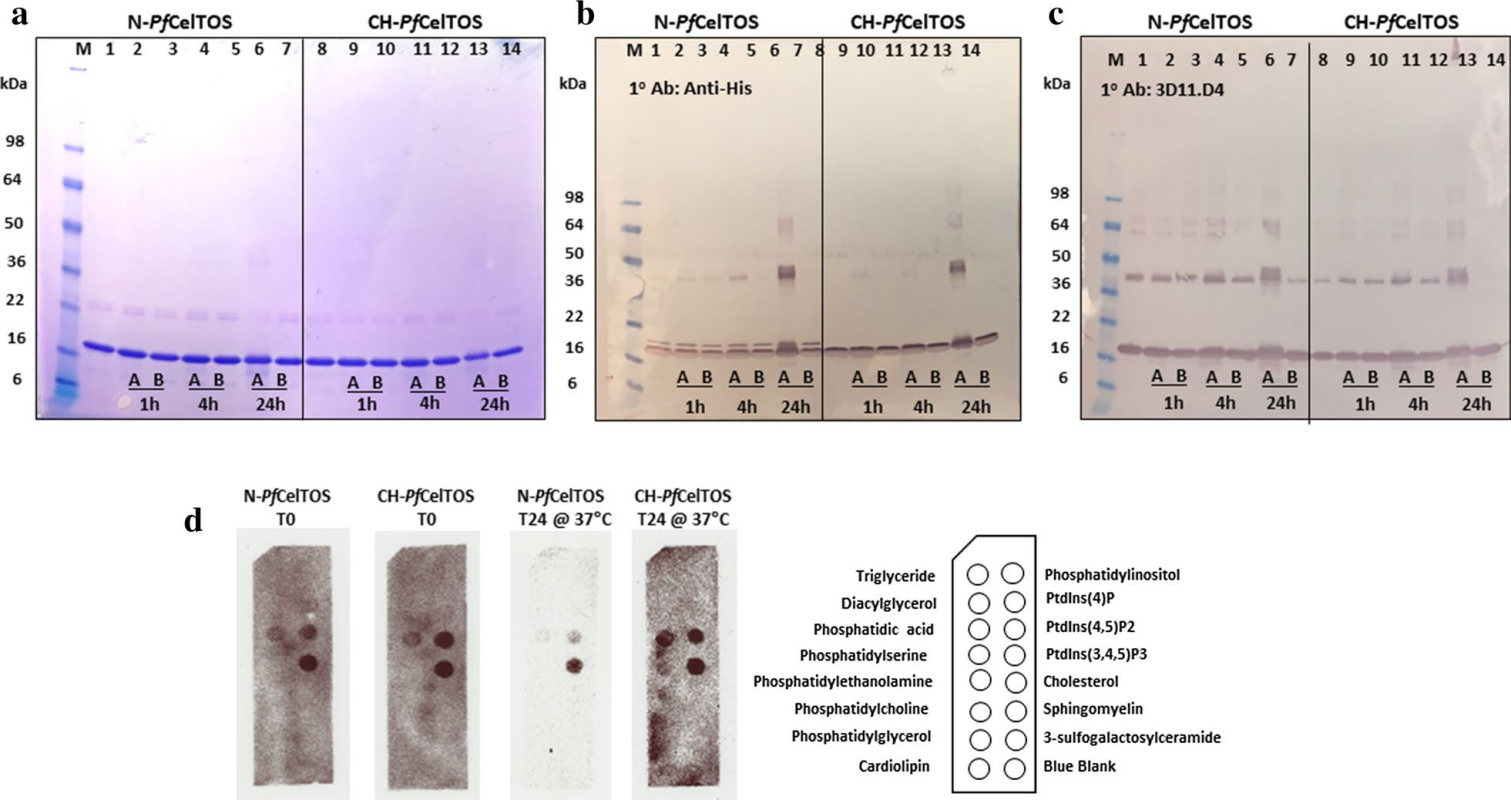
Stability of N-*Pf*CelTOS and CH-*Pf*CelTOS incubated at 37 °C and 65 °C. **a** Coomassie blue total protein staining of N- and CH-proteins incubated at A: 37 °C and B: 65 °C for 1, 4 and up to 24 h. **b** Western blot analysis using anti-histidine mAb and **c** Western blot analysis using *Pf*CelTOS-C-term specific mAb 3D11.D4. Lane 1 for each gel or blot is the reference *Pf*CelTOS protein, untreated. **d** Lipid strip analysis of N- and CH-*Pf*CelTOS incubated at 37 °C for up to 24 h. T0 indicates control lipid strips for assessing changes in binding profile of both the proteins

To address differences in protein stability, *Pf*CelTOS proteins were incubated at A: 37 °C and B: 65 °C, for 1, 4 and up to 24 h (Fig. 1). High molecular weight aggregates were observed for both proteins during the extended incubations at 37 °C when analyzed by SDS-PAGE/Western blotting (Fig. 1). Notably, this was not observed for the same proteins stored for 24 h at 65 °C, a temperature near the mid-point of the thermal denaturation curve. When probed with highly sensitive anti-His antibodies, N-*Pf*CelTOS exhibited a doublet (Fig. 1b). The weak upper band was not detected by the C-terminal *Pf*CelTOS mAb, 3D11.D4 (Fig. 1c), and was present at nominally low levels since this band was not detected by total protein staining (Fig. 1a). These results suggest that an alternative form of the protein with a slightly higher molecular weight was produced from the native sequence. This upper band was not present in the CH-*Pf*CelTOS protein. A band (~ 40 kDa) detected by western blotting in both protein preparations suggests a dimeric form of the protein that may be more resistant to denaturation. These data suggest a ‘stickiness’ that allows for some non-covalent stabilization of multimer forms. The detection of this band by both its N-terminal Histidine tag and C-terminal epitope recognizing 3D11.D4 monoclonal antibody verified that it is a form of *Pf*CelTOS and not an *E. coli* contaminant.

To assess the specific-binding characteristics of the two CelTOS proteins to cell membrane phospholipids, lipid subset spotted-arrays were evaluated. Both proteins bound to phosphatidylinositol (4,5)-diphosphate [PtdIns(4,5)P2] and phosphatidylinositol (3,4,5)-triphosphate [PtdIns(3,4,5)P3)], phospholipids residing in the plasma membrane, and to phosphatidic acid, which is present within the inner leaflet of the plasma membrane. Incubation at 37 °C for 24 h yielded significant loss of binding of N-*Pf*CelTOS to phosphatidic acid and PtdIns(4,5)P2 compared to T = 0 (Fig. 1d) while incubation at 65 °C for 24 h completely abrogated lipid binding for both proteins (data not shown). In contrast, CH-*Pf*CelTOS retained the same lipid binding characteristics as at T = 0 for 24 h at 37 °C and showed no significant functional loss in phospholipid binding. Differences in lipid binding characteristics observed at 37 °C for T = 24 h for N- and CH-*Pf*CelTOS may be attributed to the differences in their alpha helical content, the types of amino-acid misincorporations, and their propensities to aggregate after unfolding (Fig. 1d). This suggests that the CH-protein is less prone to irreversibly aggregate in solution than the N-protein as its binding site is still accessible to phospholipids at 37 °C. However, at 65 °C, a temperature near the melting temperature of the proteins, no phospholipid binding was observed for either protein at the 24 h time point (data not shown). These observations were corroborated by the western blot analysis in that at 65 °C and 24 h, the N-protein showed some level of aggregates, whereas the CH-protein had no dimers or high molecular weight multimeric aggregates (Fig. 1c).

### Protein sequence and structure analysis

**Fig. 2:**
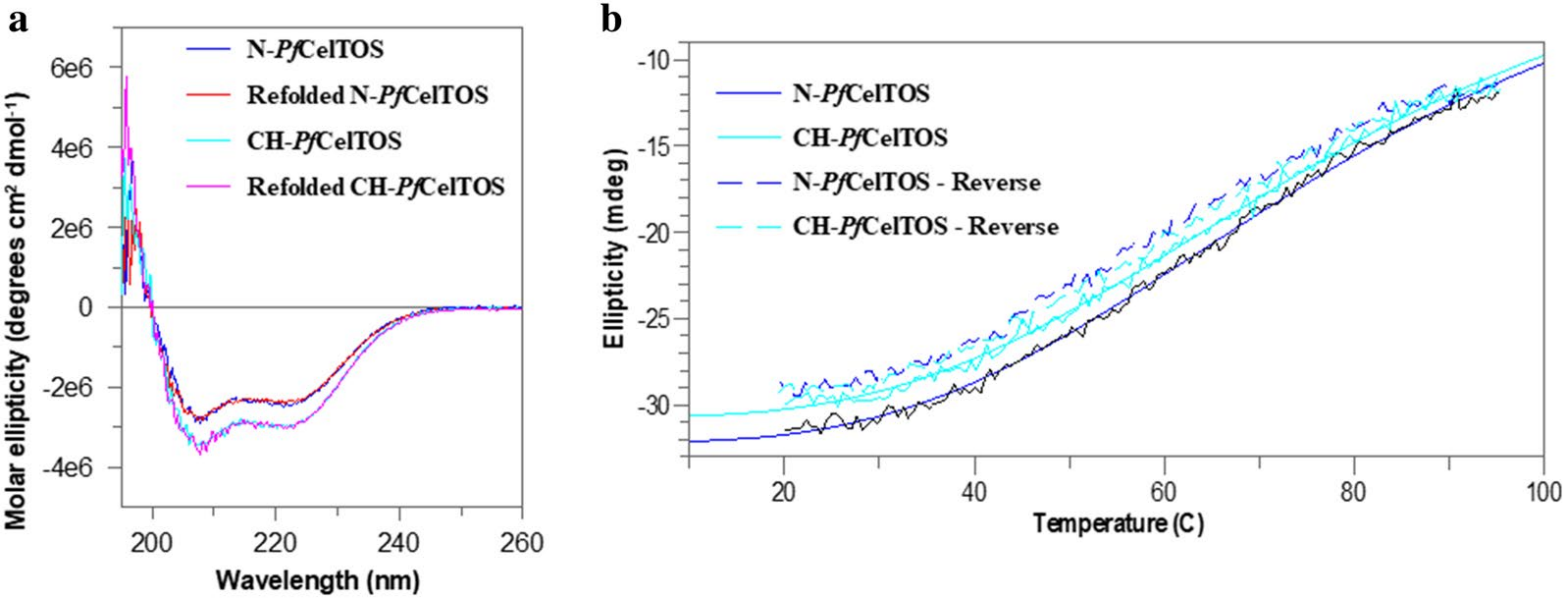
CH-*Pf*CelTOS contains more helical content than N-*Pf*CelTOS. **a** CD-spectra of N-*Pf*CelTOS (blue) or CH-*Pf*CelTOS (cyan) proteins; overlaid with the CD spectra of the refolded N-*Pf*CelTOS (red) or refolded CH-*Pf*CelTOS (pink) proteins. **b** Thermal denaturation curves of the N-*Pf*CelTOS (blue) and CH-*Pf*CelTOS (cyan) and the reversed N-*Pf*CelTOS (dashed blue) and reversed CH-*Pf*CelTOS (dashed cyan). Curves are significantly overlaid

Protein secondary structure was examined using CD spectroscopy. The structure of CelTOS from *Plasmodium vivax* (*Pv*CelTOS) shows that the protein is predominantly helical. CD scans showed that the N-*Pf*CelTOS protein had less alpha helical content than the CH-*Pf*CelTOS. The minima at 222 nm for the N-*Pf*CelTOS was 82% of that of CH-*Pf*CelTOS and correlates with the alpha helical content of the proteins (Fig. 2a). If the proteins produced from the N and CH sequences were identical no difference in alpha helical content would be expected; thus, the result is consistent with amino acid misincorporations and differences in protein primary structure. Nonetheless, despite differences in alpha helical content both proteins reversibly refolded after thermal denaturation (Fig. 2b). In contrast, a recombinant CelTOS derived from the *P. berghei* (*Pb*CelTOS) sequence showed evidence of irreversible thermal denaturation (Bergmann-Leitner et al. 2011), suggesting that only some variants of the highly conserved CelTOS protein may retain this feature and refold. For *Pb*CelTOS it is unknown as to how many amino acid misincorporations occur during expression or whether amino acid misincorporations affect its fold.

**Fig. 3:**
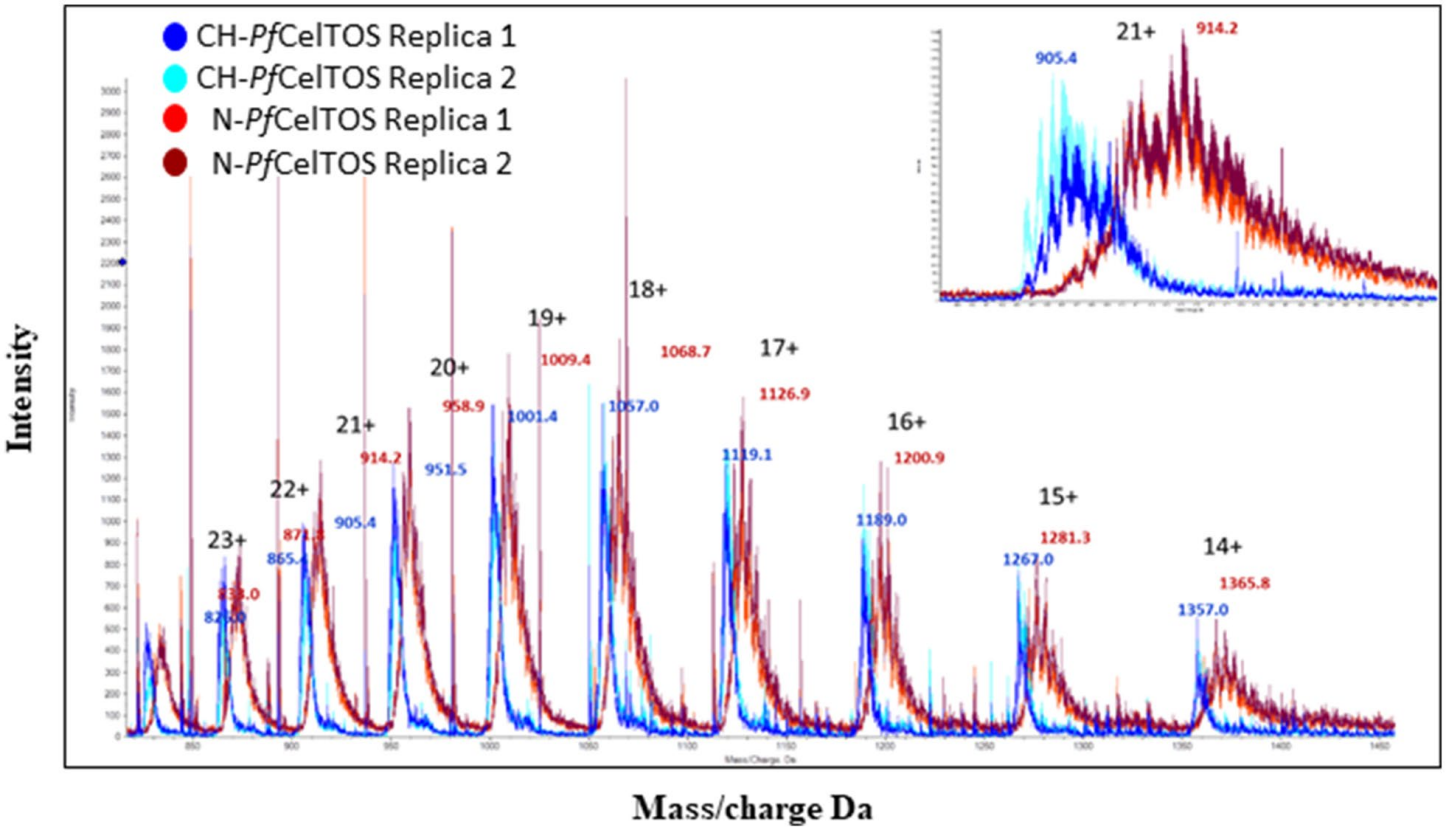
Electrospray mass spectrometry. Mass spectra of N-*Pf*CelTOS (red) and CH-*Pf*CelTOS (blue) overlaid with respective replicates (burgundy and cyan) (inset: zoomed in on 21+ charged ions)

Mass spectrometry techniques were applied to identify and quantitate the putative amino acid misincorporations in the recombinant CelTOS protein products. CH-*Pf*CelTOS had an average mass of 19,000 Da that compared well with the calculated MW of 19,027 g/mol. In contrast, the N-*Pf*CelTOS had an average mass of 19,140 Da and a broader mass/charge envelop suggesting greater heterogeneity and higher number of amino acid misincorporations compared with CH-*Pf*CelTOS (Fig. 3). This difference in the distribution of protein masses may account for the differences in secondary structure detected by CD spectroscopy. These findings indicate that the two proteins are indeed structurally different. To resolve the differences in mass detected by MS, we applied high resolution LC–MS/MS. Interestingly, amino-acid misincorporations were found in both proteins. However, non-synonymous misincorporations seen in the N-*Pf*CelTOS were more varied and numerable compared with CH-*Pf*CelTOS (Table 1). As a general observation, the most common substitutions were hydrophobic to hydrophobic followed by hydrophobic to positively charged amino acids. Hydrophobic to negatively charged amino acid substitutions or substitutions of positively or negatively charged amino acids to neutral ones were rarely observed (Table 2).

**Table 1:** Nonsynonymous amino acid substitutions observed by LC–MS/MS for N-*Pf*CelTOS and CH-*Pf*CelTOS

N-*Pf*CelTOS Amino-acid range: #1 to 80		N-*Pf*CelTOS Amino-acid range: #81 to 182		CH-*Pf*CelTOS Amino-acid range: #1 to 182	
Amino acid number, expected acid	Substitution(s)	Amino acid number, expected acid	Substitution(s)	Amino acid number, expected acid	Substitution(s)
50, S	Q	84, S	P	64. I	H
64, I	V	87, F	L	65, G	N
65, G	M/N/N	88, L	H/F	73, S	N
68, L	F	91, S	R	87, F	Y
69, A	N/H/Q	130, V	F	88, L	H
70, E	Q/Q	133, A	Q	144, S	R
71, T	N/H/Q	135, Y	H/F		
72, I	M	137, I	M/V/V		
75, E	D	138, I	M/V/T/M/M		
83, N	Y	139, V	R

**Table 2:** Nature of amino-acid substitutions for N-*Pf*CelTOS and CH-*Pf*CelTOS

Type of substitution	Amino acid number: N-*Pf*CelTOS	Amino acid number: CH-*Pf*CelTOS
Hydrophobic to hydrophobic	64, 68, 72, 87, 88, 130, 135, 137, 137, 137, 138, 138, 138, 138	87
Hydrophobic to (+) charged	69, 88, 135, 139	64, 88
Hydrophobic to (−) charged	None observed	None observed
Neutral to hydrophobic	83	None observed
Hydrophobic to neutral	69, 69, 133, 138	None observed
(+) charged to neutral	None observed	None observed
(−) charged to neutral	70	None observed
Polar uncharged to polar uncharged	50, 71, 71	73
Polar uncharged to (+) charged	71, 88, 91	144
Gly/Pro/Ser to hydrophobic	65	None observed
Gly/Pro/Ser to polar uncharged	50, 50	65
Gly/Pro/Ser to Gly/Pro/Ser	84	None observed

**Fig. 4:**
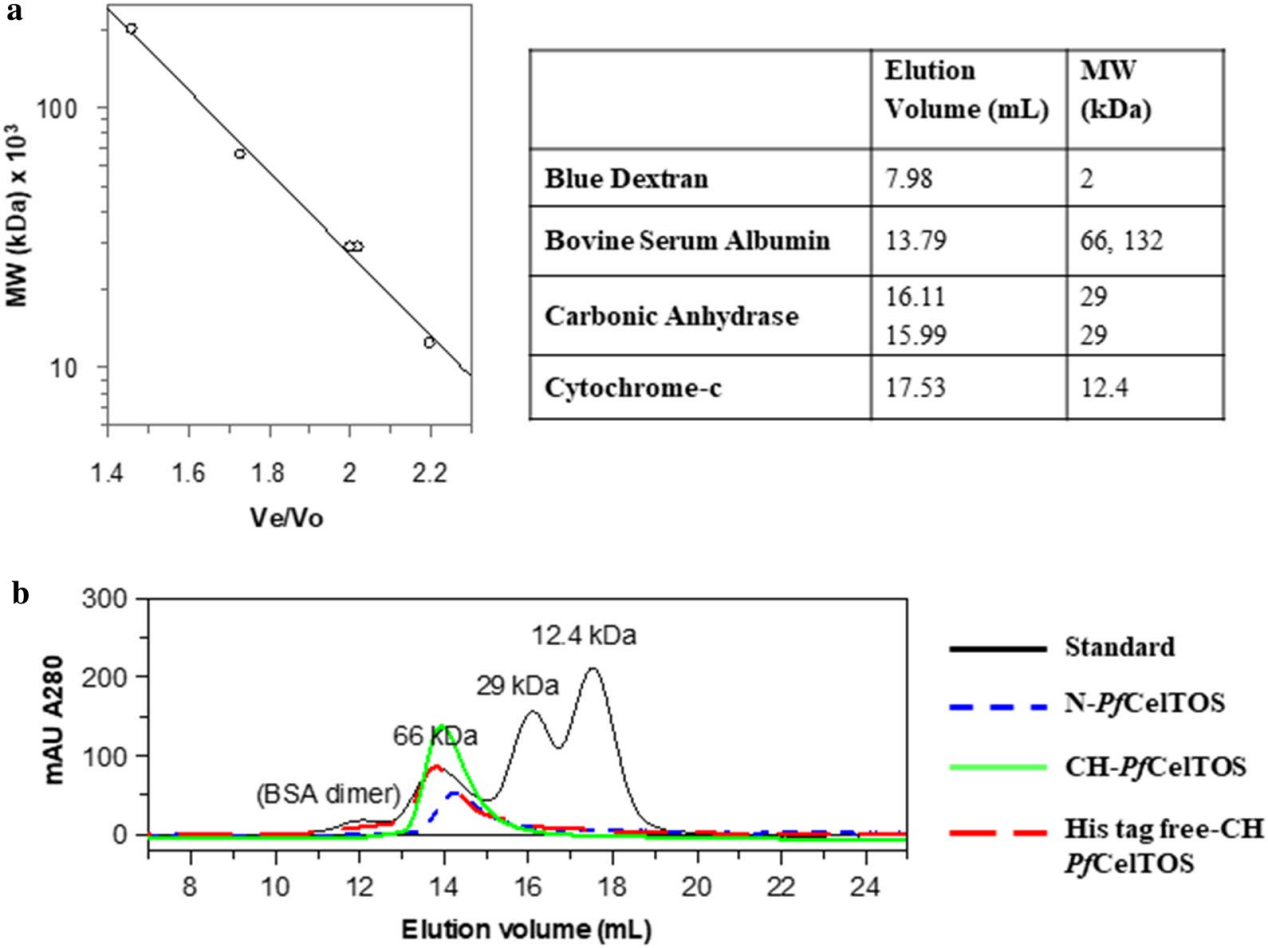
Size-exclusion chromatography. **a** Calibration curve using calibration standards: blue dextran (2 kDa), bovine serum albumin (66 kDa), carbonic anhydrase (29 kDa), and cytochrome-*c* (12.4 kDa). **b** N-*Pf*CelTOS is shown in blue dash, CH-*Pf*CelTOS is shown in green and a histidine tag-free version of CH-*Pf*CelTOS is shown in red dash. Calibration standards are overlaid and shown in black

**Fig. 5:**
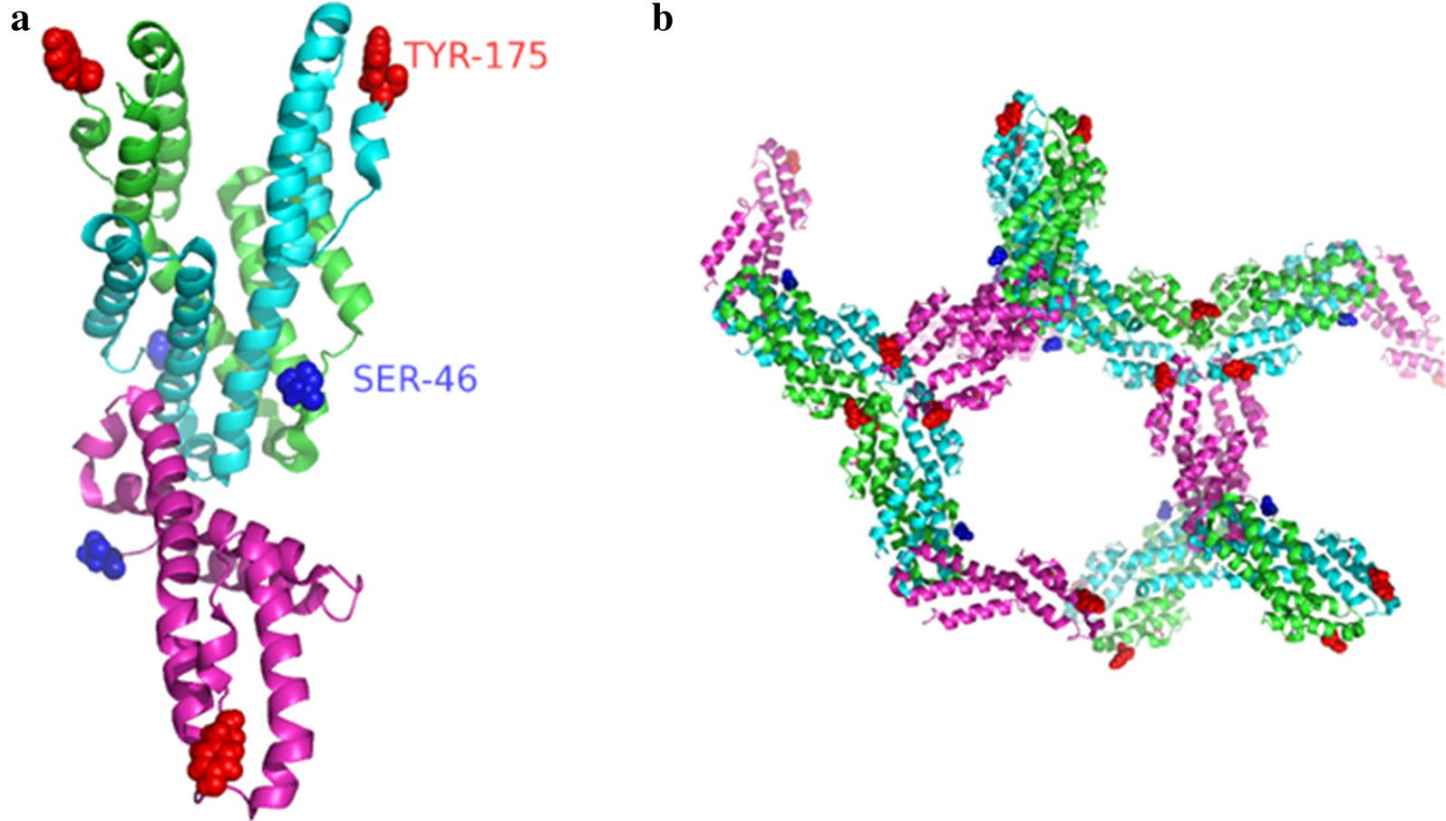
Structure of CelTOS (PDB 5TSZ) from *P. vivax.* **a** Three molecules (green, cyan and magenta) are found in the unit cell. **b** Crystal packing is shown. The first and the last ordered residues are Ser-46 (blue spheres) and Tyr-175 (red spheres). The structure is predominantly helical

To determine if codon usage affected quaternary structure, a calibrated gel-filtration column was used. The column was calibrated using three standard proteins (66, 29, 12.4 kDa) and blue dextran to estimate the void volume. The data were fit to a single exponential decay equation (A = A_0_ * e^−kr^) where v = elution volume of the protein, v_o_ = void volume, r = v/v_o_, and A = MW (kDa). From a fit to our data, we obtained an A_0_ = 38,342 and k = 3.6245 and were able to calculate the MW of the *Pf*CelTOS proteins produced using the native or codon-harmonized sequence. For this analysis, we compared three proteins: (1) N-*Pf*CelTOS; (2) CH-*Pf*CelTOS; and (3) a histidine tag-free version of the CH-*Pf*CelTOS, all produced in *E. coli*. The N-*Pf*CelTOS eluted as a 59 kDa (14.3 mL) protein while the CH-*Pf*CelTOS eluted at 70 kDa (13.9 mL). The histidine tag-free CH-*Pf*CelTOS eluted at 73 kDa (13.8 mL). Notably, in the histidine tag-free CH-*Pf*CelTOS chromatogram, a second, relatively broad peak eluted between 12 and 13 mL, correlating with a MW range of ~ 104–166 kDa. This peak may correspond to a histidine tag-free hexamer (104 kDa) or octamer (139 kDa). The ~ 70–73 kDa peaks overlaid well with the 66 kDa standard and may indicate a tetramer (70–76 kDa) depending upon the relative globularity between the multimer and standards (Fig. 4). N-*Pf*CelTOS eluted near where a trimer was expected (57 kDa). Notably, monomers were not observed in either sample. Interestingly, the quaternary structure of a C-terminal His-tagged *P. vivax* CelTOS (*Pv*CelTOS) was previously reported to be a homodimer based upon analytical ultracentrifugation data (Jimah et al. 2016). However, three molecules of the protein are found within the asymmetric unit of the crystal, and symmetry-related molecules in the crystal lattice pack as hexamers with no density for the N-terminal residues prior to Ser-46 or for the C-terminal residues beyond Tyr-175 (PDB 5TSZ) (*Pv*CelTOS amino acids 36–196) suggesting that they are either disordered or absent (i.e., degraded or proteolyzed) (Jimah et al. 2016) (Fig. 5). Thus, other quaternary structures cannot be excluded. Since epitopes can form at interfacial locations in multimeric proteins, we next evaluated immunogenicity in mice.

### Immunity in mice

**Fig. 6:**
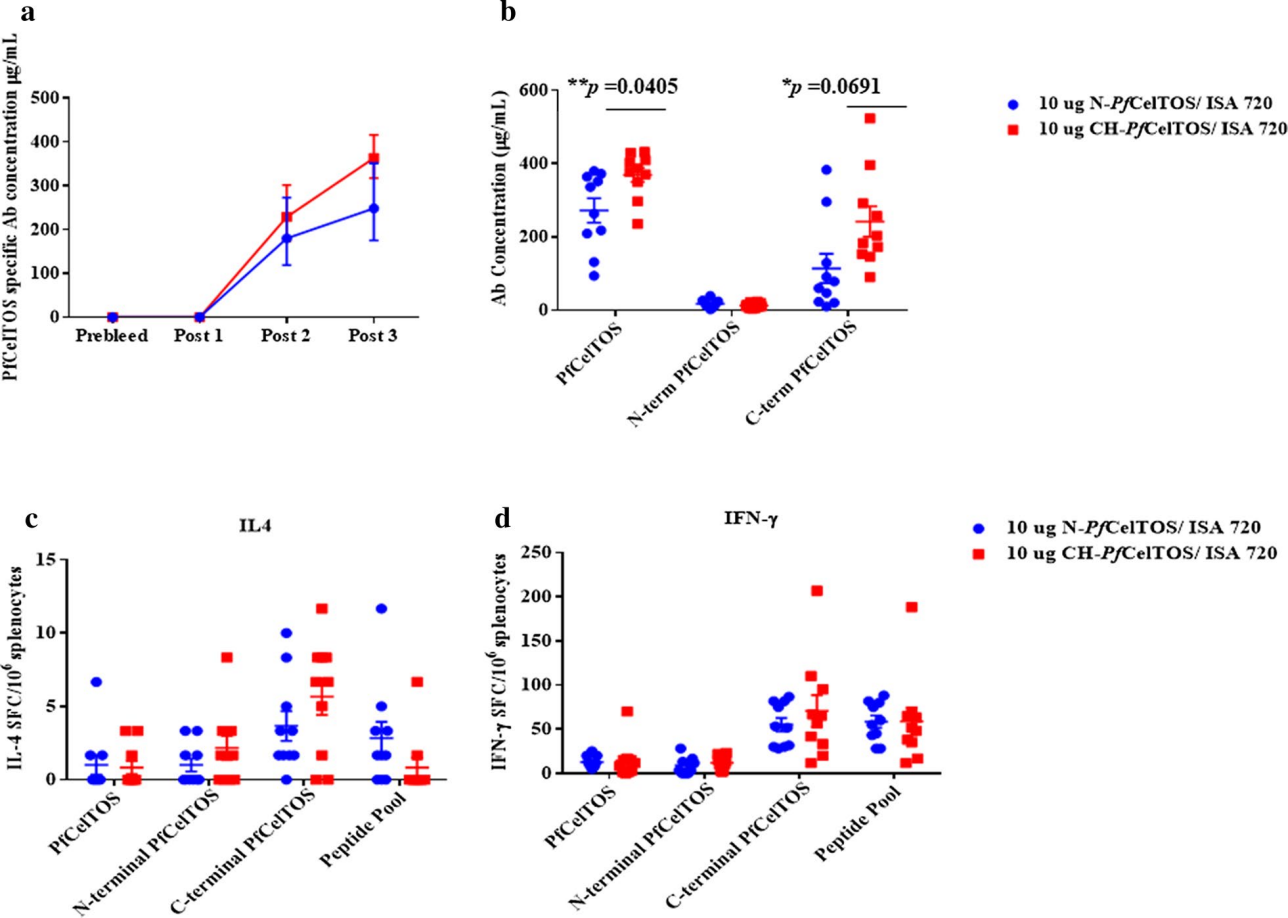
N-*Pf*CelTOS and CH-*Pf*CelTOS induce comparable antibody and TH1 biased cellular responses. **a** Kinetic of *Pf*CelTOS specific antibody responses, reported as antigen specific concentrations (µg/mL). Geometric means and 95% confidence interval are reported. **b** Scatter plot of individual mouse responses at 2 weeks post 3rd immunization reported as the mean and standard deviation. Statistical significance of differences in antibody responses induced by N-*Pf*CelTOS and CH-*Pf*CelTOS was determined using Parametric, two tailed, unpaired T-test (**p = 0.0214, *p = 0.00393 against *Pf*CelTOS and C-terminus of *Pf*CelTOS proteins, respectively). Splenocytes from individual mice were tested for secretion of IL-4 and IFN-γ cytokines by ELISpot. Data are reported as the mean plus standard error of the mean of cytokine producing cells (spot forming cells (SFC)/10^6^ splenocytes) of individual mice (**c**) IL-4 and (**d**) IFN-γ

To evaluate immune responses induced by N-*Pf*CelTOS and CH-*Pf*CelTOS, inbred Balb/cJ mice were immunized either with 10 µg of N-*Pf*CelTOS or CH-*Pf*CelTOS in Montanide ISA 720 (n = 10/group). Antibody concentrations were determined using a standard ELISA. Serum antibodies were tested against full-length CH-*Pf*CelTOS, N-terminal CH-*Pf*CelTOS and C-terminal CH-*Pf*CelTOS recombinant proteins to assess antibody fine specificities. Both N-*Pf*CelTOS and CH-*Pf*CelTOS immunogens induced robust antibody responses after three immunizations. Antibodies to the full-length and the C-terminal portion of *Pf*CelTOS were detected in high proportions, and statistically higher for the CH-*Pf*CelTOS immunogen (Unpaired t-test, p = 0.0214 and p = 0.0393, respectively), while N-terminal *Pf*CelTOS recognition was marginal for both groups (Fig. 6b). For cellular responses, the frequencies of IL-4 and IFN-γ-producing splenocytes were measured by ELISpot (Bergmann-Leitner et al. 2010). *Pf*CelTOS-specific IFN-γ secreting splenocytes were detected in high numbers, indicating a Th1 skew for both groups (Fig. 6c), with significant ex vivo stimulation using the C-terminal *Pf*CelTOS and *Pf*CelTOS peptide pool compared to the full-length or N-terminal CH-*Pf*CelTOS proteins. Differences in stimulation indices for the shorter C-terminal *Pf*CelTOS protein and the peptide pool compared with the full length *Pf*CelTOS may be partially explained by better processing and improving antigen presentation and T‐cell activation. The lack of stimulation with the full length protein for detection of IFN-γ secreting splenocytes was nominally unexpected (Bergmann-Leitner et al. 2010) and may reflect the quality of the stimulating antigen following multiple freeze thaw cycles.

## Discussion

Single synonymous codon substitutions within a coding region can significantly alter substrate specificities (Kimchi-Sarfaty et al. 2007) or enzymatic activities (Komar et al. 1999) of proteins demonstrating that even subtle changes can have a significant impact on final protein structure/function. These subtle differences in coding sequence may serve to regulate protein folding kinetics. While we and others have observed that codon substitutions can alter protein expression, primary, secondary, and quaternary structure, the impact of these changes has not been fully assessed for vaccine immunogens. For example, altered peptide ligands have been intentionally used to modulate T-cell responses to immunotoxin therapeutics (Candia et al. 2016; Castelletti and Colombatti 2005). The so-called Hoskins effect (also known as “original antigenic sin”) refers to a phenomenon where prior exposure to an antigen can alter immune responses to a second similar antigen and reduce an immune response. Thus, we hypothesized that variability in amino acid sequence could potentially impact immune responses. Here, *Pf*CelTOS was “codon harmonized” to optimize its expression. It was then systematically compared with protein expressed from the native DNA sequence to evaluate the impact of codon usage on the integrity of the final product. Our findings suggest that the two proteins are biophysically different in primary, secondary, and quaternary structure.

In comparing N-*Pf*CelTOS and CH-*Pf*CelTOS, we expected to find differences in expression levels, solubility, and yield for the two products as previously observed for MSP1_42_ (Khan et al. 2012). While both proteins expressed well in *E. coli* and were highly soluble, we found them to be different in mass, homogeneity (number of misincorporation events), and secondary structure. Differences in quaternary structure also were detectable in size exclusion chromatograms. Notably, both *Pf*CelTOS proteins reversibly refolded after thermal denaturation; thus, any differences in secondary structure did not significantly affect reversible refolding.

We found that the codon-harmonized protein mass was more similar to the theoretical molecular weight, compared with the native protein which exhibited a greater mass. The broader distribution of masses in mass spectra suggested that the protein produced from the native DNA sequence was more heterogeneous. An attempt to identify the cause of these differences in mass revealed a significantly higher number of amino acid misincorporations in protein expressed from the native DNA sequence. Although amino acid misincorporation is reported to occur at low levels in *E. coli*, it is dependent upon many factors including the expression system and cell lines (Bouadloun et al. 1983; Edelmann and Gallant 1977; Ellis and Gallant 1982; Kramer and Farabaugh 2007; Laughrea et al. 1987; Loftfield 1963; Loftfield and Vanderjagt 1972; Manickam et al. 2014; Stansfield et al. 1998; Yu et al. 2009; Zhang et al. 2013). Generally, it is estimated that anywhere between 10 and 50% of misincorporations affect protein function (Drummond and Wilke 2008, 2009). These sequence variations can cause protein heterogeneity and altered catalytic activity (Kramer and Farabaugh 2007; Stansfield et al. 1998), disrupt ligand and substrate binding and affect protein folding, leading to aggregation (Drummond and Wilke 2008; Lee et al. 2006). In some cases, high levels of sequence variants can cause undesired immune responses (Drummond and Wilke 2008, 2009; Katsara et al. 2008). Interestingly, misincorporation at rarely used codons does not occur more frequently than at commonly used codons, rather misincorporation errors may be context-dependent (Parker 1992). In the current study, we observed a larger number of sequence variations in N-*Pf*CelTOS than in the CH-*Pf*CelTOS. The most common substitutions being hydrophobic to hydrophobic amino acids, and hydrophobic to positively-charged amino acids. Interestingly, hydrophobic to negatively-charged amino acid substitutions and positively-charged to neutral substitutions were not observed. These substitutions likely account for the differences in alpha helical content and suggest that codon usage impacts the misincorporation of amino acids. A factor not extensively discussed is the influence of the Histidine-tag and its location on protein tertiary and quaternary structure, as well as its influence on immunogenicity. A codon-optimized, C-terminal His-tagged *Pv*CelTOS (*P. vivax* CelTOS) was previously shown to form a homodimer (Jimah et al. 2016); our results indicate that the N-terminal His-tagged or a tag-free, codon harmonized *Pf*CelTOS appears to form multimers larger than a dimer (possibly trimers or tetramers). These predictions of higher order structures are of particular interest with regards to the role of *Pf*CelTOS in host cell traversal. It has been shown that CelTOS binds to phosphatidic acid on the inner leaflet of the cell membrane and functions to disrupt the plasma membrane by assembling a pore on the cytoplasmic-face to enable the exit of parasites from invaded host cells during cell traversal (Jimah et al. 2016). A biologically relevant in vivo phenomenon requiring protein functionality, nominally at an elevated temperature of 37 °C (Fig. 1d).

Quaternary structure can affect the immunogenicity of subunit vaccines in cases where conformational epitopes are present at protein–protein interfaces. For example, antibodies against viral envelop proteins can bridge adjacent epitopes to prevent conformational changes and can ultimately inhibit entry and egress of enveloped viruses (Fox et al. 2015). With respect to immunogens, the presentation of mixtures of different but related protein sequences such as site-directed mutants can reduce humoral immune responses and can impact the protective efficacy of an immunogen (Candia et al. 2016). These immunosuppressive effects are sequence-dependent as only some peptides bind MHC molecules and T-cell receptors. Here, we did not observe a significant difference in cellular responses; however, antibody levels induced by the CH-*Pf*CelTOS were significantly higher than those induced by N-*Pf*CelTOS. Accurate detection of the effects of low abundance mistranslated proteins and peptides, against a background of wild-type protein, is challenging. Our findings suggest that the impact of these misincorporations on the immunogenicity of these protein products may be relatively low. Nevertheless, the impact of these sequence variants on the final product quality is difficult to estimate. Differences in the loss of phospholipid binding affinity were observed between the N-*Pf*CelTOS and CH-*Pf*CelTOS after the protein was subjected to a 24 h incubation at 37 °C suggesting a reduced propensity for the CH-protein to irreversibly aggregate. It should be noted that the recombinant *Pf*CelTOS constructs used here do not have cysteine residues. The CH-protein better retained its ability to bind phospholipids after the 37 °C 24 h incubation, suggesting a level of structural integrity. High molecular weight multimers that withstood SDS-PAGE separation also were observed for both proteins in western blots after 24 h incubation at 37 °C. After incubation at 65 °C for 24 h, a temperature near the mid-point of the thermal denaturation curve, fewer dimers and high molecular multimers were observed for the CH-protein in western blots suggesting fewer non-specific aggregates. Aggregation and protein precipitation are typically irreversible processes. Thus, these differences in thermostability may affect long-term storage. While heterologous expression in *E. coli* may be facile and economical, our results show the potential impact of codon usage on the fidelity of protein synthesis and protein homogeneity.

## Data Availability

Accession number, KH833194 identified the nucleic acid sequence of the CH-*Pf*CelTOS. All materials are available upon request.
